# Ghrelin mediated cardioprotection using in vitro models of oxidative stress

**DOI:** 10.1038/s41434-023-00435-9

**Published:** 2024-01-04

**Authors:** Cindy Y. Kok, George Ghossein, Sindhu Igoor, Renuka Rao, Tracy Titus, Shinya Tsurusaki, James JH. Chong, Eddy Kizana

**Affiliations:** 1 Centre for Heart Research, The Westmead Institute for Medical Research, The University of Sydney, Sydney, NSW Australia; 2 Westmead Clinical School, the Faculty of Medicine and Health, The University of Sydney, Sydney, NSW Australia; 3 Department of Cardiology, Westmead Hospital, Westmead, NSW Australia

**Keywords:** Cardiovascular diseases, Gene therapy

## Abstract

Ghrelin is commonly known as the ‘hunger hormone’ due to its role in stimulating food intake in humans. However, the roles of ghrelin extend beyond regulating hunger. Our aim was to investigate the ability of ghrelin to protect against hydrogen peroxide (H_2_O_2_), a reactive oxygen species commonly associated with cardiac injury. An in vitro model of oxidative stress was developed using H_2_O_2_ injured H9c2 cells. Despite lentiviral ghrelin overexpression, H9c2 cell viability and mitochondrial function were not protected following H_2_O_2_ injury. We found that H9c2 cells lack expression of the preproghrelin cleavage enzyme prohormone convertase 1 (encoded by *PCSK1*), required to convert ghrelin to its active form. In contrast, we found that primary rat cardiomyocytes do express *PCSK1* and were protected from H_2_O_2_ injury by lentiviral ghrelin overexpression. In conclusion, we have shown that ghrelin expression can protect primary rat cardiomyocytes against H_2_O_2_, though this effect was not observed in other cell types tested.

## Introduction

Reactive oxygen species (ROS) are critical signalling molecules which play an important role in physiological cellular signalling and function in the heart. These molecules are tightly regulated by endogenous antioxidants, to maintain intracellular redox homoeostasis. When ROS are at levels that exceed antioxidant capacity, oxidative stress occurs. This can then lead to apoptosis and pathogenesis of cardiovascular disease. Hydrogen peroxide (H_2_O_2_), the free radical superoxide anion (O^2−^) and the hydroxyl radical (OH^−^) are the main oxygen species that cause oxidative stress.

Augmenting antioxidant activity in the heart has been shown to protect against the deleterious effects of oxidative stress in cardiomyocytes. General antioxidant therapies include antioxidant inflammation modulator drugs, superoxide dismutase/catalase combinations or vitamin E analogues, though these have had limited success in clinical translation in patients with cardiovascular disease [1–4]. Naturally occurring antioxidants such as resveratrol and quercetin have also shown promise in pre-clinical models of ischaemia/reperfusion, though this therapeutic benefit was not consistently observed in clinical studies [5–8]. Currently, drugs such as allopurinol or oxypurinol are used to treat patients with heart failure by inhibition of xanthine oxidase, with varied efficacy [9–11]. Therefore, there is a need to explore alternative molecules with more consistent cardioprotective activity.

Ghrelin is a 28 amino acid peptide hormone primarily produced in the oxyntic glands of the stomach [12], that has emerged as a cardioprotective peptide with evidence of anti-inflammatory, antioxidant and anti-apoptotic effects in myocardial and endothelial cells [13]. Physiological actions of ghrelin in the heart include increasing cardiac output and modulating the electrical activity by supressing cardiac sympathetic nerve activity and stimulating cardiac parasympathetic nerve activity [14]. Besides these physiological actions, ghrelin has been shown to have cardioprotective effects in multiple studies.

In a 2011 study, ghrelin protein was administered to H9c2 cells, a rat cardiac myoblast clonal cell line, and shown to decrease H_2_O_2_ induced apoptosis in a dose dependent manner [13]. Further, in a 2013 study, ghrelin protein was shown to protect primary rat neonatal cardiomyocytes from dithiothreitol injury by reducing apoptosis [15]. Ghrelin administration via an adeno-associated viral vector was shown to preserve cardiac function in a myocardial infarct mice model by increasing autophagy [16]. Importantly, ghrelin has had beneficial effects in patients with chronic heart failure after intravenous administration [17]. A beneficial effect was also seen in a clinical trial where patients with HFrEF received intravenous infusion of the recombinant protein, with increased cardiac output in the absence of hypotension, tachycardia, arrhythmia or ischaemia [18]. However, the mechanism of ghrelin mediated cardioprotection is not fully elucidated clinically.

Most studies exploring the protective role of ghrelin utilise recombinant ghrelin protein which requires multiple doses of administration due to the short half-life of ghrelin which is approximately 30 min [19]. For long term benefit, it would be ideal to induce cardioprotection with a treatment which only requires one administration. Hence, in this study, we utilised a lentiviral vector to administer ghrelin. As the lentiviral vector transduces cells, the provirus encoding the gene of interest, in this case ghrelin, is incorporated into the host cell genome, leading to permanent expression [20]. This means that compared to utilising recombinant ghrelin protein, lentiviral vector ghrelin gene therapy requires only a single dosage of administration.

**Fig. 1:**
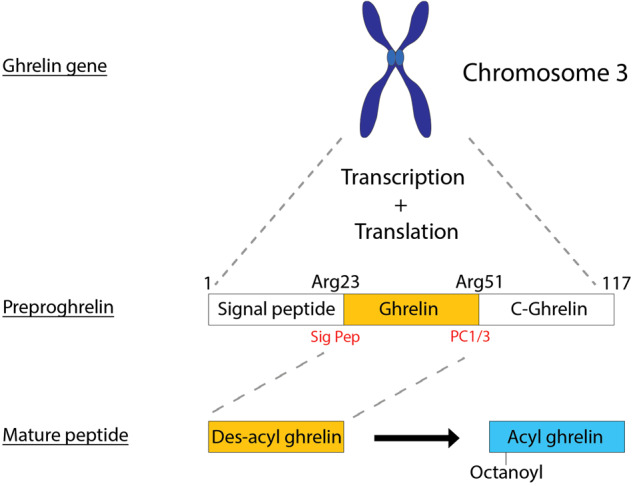
Processing of the hormone ghrelin. The ghrelin gene located on chromosome 3 is initially transcribed and translated into preproghrelin (117 amino acids). Preproghrelin is then cleaved by the enzymes signal peptidase (Sig Pep) and prohormone convertase 1/3 (PC1/3) encoded by *PCSK1* (at arginine 51) to form mature ghrelin (28 amino acids). Adapted from [34].

The ghrelin gene is located on chromosome 3 and is initially transcribed and translated into the prohormone preproghrelin, which is 117 amino acids (Fig. 1). Preproghrelin is then cleaved by the enzymes signal peptidase and prohormone convertase 1/3 (PC1/3) to form mature ghrelin (Fig. 1). Therefore, to observe the cardioprotective effects of gene therapy mediated ghrelin expression, it is important to design the therapeutic gene cassette and/or to transduce target cells which are able to produce the active form of ghrelin via correct post-translational modification.

We sought to determine the utility of ghrelin gene therapy to protect against oxidative stress initially in H9c2 cardiac myoblasts. Further studies were then performed using neonatal rat ventricular myocytes (NRVMs) and human induced pluripotent stem cell derived cardiomyocytes (hiPSC-CMs), to determine the downstream effects on cell viability and formation of cardioprotective ghrelin.

## Methods

### Cell culture

The cardiac-derived myoblast cell line, H9c2 cells (Sigma-Aldrich, #88092904-1VL, St. Louis, MO, USA) and HEK293T cells (ATCC, CRL-3216^TM^, Manassas, VA, USA) were maintained in Dulbecco’s Modified Eagle Medium (DMEM) (Lonza, #12-604F, Basel, Switzerland) supplemented with 10% FBS (v/v) (Gibco, #1099-141, Massachusetts, USA) and 1% L-glutamine (v/v) (Sigma-Aldrich, #G7513-100ML, St. Louis, MO, USA) at 37 ^o^C in a humidified atmosphere containing 5% (v/v) CO_2_ cells.

### Neonatal rat ventricular myocytes

All animal procedures had ethical approval (Western Sydney Local Health District animal ethics protocol 4332) and were performed in accordance with the National Health and Medical Research Council (NHMRC) Code for the care and use of animals for scientific purposes. NRVMs were isolated from day 3 (D3) neonatal Wistar rats via enzymatic dissociation of ventricles using trypsin and collagenase as previously described [21]. Isolated NRVMs were seeded at 2–4 × 10^5^ cells/well in 24 well plates. Twenty-four hours later, cells were washed with DPBS (Lonza, #12001-664, Basel, Switzerland). On D2 after cell plating, FBS was reduced to 2% and media was changed on cultures every second day.

### Human induced pluripotent stem cells

The hiPSC line SCVI 8 used in this study was obtained from Professor Joseph Wu (Stanford Cardiovascular Institute, USA). Stem cells were maintained on Matrigel (Corning, #354277, New York, USA) coated 60 mm cell culture dishes using the mTeSR^TM^ Plus kit (STEMCELL Technologies, #05825, Vancouver, Canada). Upon confluence, cells were passaged as colonies using gentle cell dissociation reagent (STEMCELL Technologies, #07174, Vancouver, Canada) every 6-7 days.

For cardiomyocyte differentiation, cells were dissociated from confluent dishes on D-2 using TrypLE^TM^ Express Enzyme (ThermoFisher Scientific, 12604-021, Massachusetts, USA), then seeded into Matrigel coated 12 well plates at 7 × 10^5^ cells/well using mTeSR^TM^ Plus supplemented with Y-27632 (STEMCELL Technologies, #72304, Vancouver, Canada). On D-1, a media change was performed to remove the Y-27632. On D0, the differentiation was commenced for both the STEMdiff (SD) kit method and the Small Molecule (SM) method. For the SD method, differentiation was commenced using the STEMdiff Cardiomyocyte Differentiation Kit (STEMCELL Technologies, #05010, Vancouver, Canada) according to manufacturer’s instructions until the point of maintenance in STEMdiff Cardiomyocyte Maintenance Medium (CMM). Cells were maintained on CMM until differentiation completion on D15. For the SM method, differentiation was commenced by addition of 10 μM CHIR-99021 (Tocris Bioscience, #4423, Bristol, United Kingdom), 1x B27-Insulin supplement (Life Technologies, #A1895601, California, USA) and 1x penicillin-streptomycin solution (Life Technologies, #15140122, California, USA) in RPMI 1640 (Life Technologies, #21870092, California, USA). The next day, the media was replaced with RPMI containing 1x B27-Insulin and 1x penicillin-streptomycin solution. On D3, the media was exchanged for RPMI containing 1x B27-Insulin, 1x penicillin-streptomycin and 5 μM IWP-2 (Tocris Bioscience, #3533, Bristol, United Kingdom). On D5, the media was exchanged for RPMI containing 1 x B27-Insulin and 1x penicillin–streptomycin without supplemental cytokines. From D7 onwards, the cultures were fed every 2 days with RPMI plus 1x B27 supplement (Life Technologies, #17504001, CA, USA) and 1x penicillin–streptomycin until differentiation completion on D15.

Once differentiation was finished, cells were replated for further experiments. After 1 h of incubation with 10 µM Y-27632, differentiated cardiomyocytes were dissociated using TrypLE^TM^ Express supplemented with 2 µg/mL DNase I (STEMCELL Technologies, #07900, Vancouver, Canada). Dissociated cells were then replated into Geltrex^TM^ (Gibco, #A14133-02, Massachusetts, USA) coated 24 well plates at 5 × 10 ^5^ cells/well using RPMI 1640 media and B27 supplement accompanied with Y-27632.

### Molecular cloning

A gene block was synthesised (Integrated DNA Technologies Pte. Ltd, Singapore, Republic of Singapore) to contain the human ghrelin sequence (Genbank: BC025791.1). The ghrelin cDNA (full length preproghrelin) was cloned into the lentiviral vector plasmid pRRLsin18.cPPT.CMV.GFP.Wpre (ppt.CMV.GFP, Inder Verma, The Salk Institute for Biological Studies, California, USA) after removal of GFP. The new vector was named ppt.hGhre. A control vector was also generated, by including LacZ coding sequence in place of the ghrelin transgene (ppt.LacZ).

A truncated “minighrelin” vector (ppt.mini.Ghre) was produced in an attempt to circumvent the requirement for PC1/3 mediated cleavage of preproghrelin into the mature ghrelin peptide. The proximal 150 bp of the ghrelin coding sequence was cloned with an in-frame stop codon into the lentiviral vector plasmid, as described for the ppt.hGhre vector (see also Supplementary Fig. 3).

### Lentiviral vector production

Lentiviral vectors were produced by calcium phosphate transfection into HEK293T cells. Vector containing supernatant was collected at 48 and 72 h after transfection, then filtered and concentrated by ultrafiltration (100,000 MWCO PES, sartorius, #VS2042, Gottingen, Germany). Concentrated virus (LV.LacZ, LV.miniGhre and LV.hGhre) was aliquoted and stored at −80 °C. Transduction titre was assigned on concentrated supernatant by assessing transgene expression in HEK293T cells using a limiting dilution assay in the presence of polybrene 8 μg/mL (Sigma-Aldrich, #H9268-10G, St. Louis, MO, USA) four days after transduction. For transduction experiments, concentrated vector stock was used at the indicated multiplicity of infection (MOI) in the presence of polybrene 8 μg/mL. Vector was applied for 18 h, and media changed to 2% FBS in DMEM the following morning.

### Western blots

For analysis of intracellular ghrelin, protein was extracted from cell pellets using RIPA buffer and 25x protease inhibitor (Sigma-Aldrich, #P8340-1ML, St. Louis, MO, USA), then quantified using the Pierce™ BCA Protein Assay Kit (ThermoFisher Scientific, #23227, Massachusetts, USA). Protein samples were diluted in 4x laemmli sample buffer, denatured at 95 °C for 5 min, then loaded onto a Mini-PROTEAN® TGX™ Precast Gel (Bio-Rad, #4561094, CA, USA), along with a 10–250 kDa protein ladder, with SDS-PAGE at 100 V for 1 h in 1× running buffer. Proteins were transferred onto a nitrocellulose membrane in 1X transfer buffer, using a Mini Trans-Blot® Electrophoretic Transfer Cell (Bio-Rad, #1703930, CA, USA) at 70 V for 1.5 h.

Afterwards, the membrane was temporarily stained with Ponceau S to observe the total protein transferred to the membrane. The membrane was then blocked for 1 h in 5% skim milk, followed by overnight incubation with the primary mouse anti-ghrelin (MERCK Millipore, MAB10404, New Jersey, USA) antibody. The membrane was subsequently washed 3 × 10 min with PBST, followed by incubation with the rabbit anti-mouse (Sigma-Aldrich, #A9044-2ML, St. Louis, MO, USA) secondary antibody for 1 h. The membrane was then washed 3 × 10 min with PBST then imaged via chemiluminescence using the SuperSignal™ West Femto Maximum Sensitivity Substrate (ThermoFisher Scientific, #34095, Massachusetts, USA). The membrane was stripped with 0.5 M NaOH, then incubated with a rabbit anti-β-actin antibody (abcam, #ab8227, Massachusetts, USA), goat anti-rabbit secondary antibody and imaged as previously described. Immunoblots were quantified using ImageJ.

To determine whether biologically active mature ghrelin was being secreted into the supernatant of H9c2 cells transduced with LV.hGhre or LV.miniGhre, 12 mL of supernatant was collected from transduced cells and concentrated using a Vivaspin 20 (3000 MWCO PES, sartorius, #VS2091, Gottingen, Germany) at 3500 × *g* and 4 °C for 15 min (or until approximately 200 µL of supernatant remained). A Western blot was performed on this concentrated supernatant as previously described.

### Amplification of ghrelin, PSCK1 and GAPDH transcript by RT-PCR

RNA was extracted from H9c2 cells, NRVMs and U87-MG cells using the ISOLATE II RNA Mini Kit (Bioline, #BIO-52072, Tennessee, USA) then quantified using a NanoDrop™ 2000 Spectrophotometer (ThermoFisher Scientific, #ND2000, MA, USA). For cDNA synthesis, Random Primers (500 ng/μL) were added to the extracted RNA (1 μg), followed by incubation at 70 °C for 5 min. M-MLV Reverse Transcriptase Buffer (5×), M-MLV Reverse Transcriptase (Promega, #M1701, Wisconsin, USA), 10 mM dNTPs (New England Biolabs, #N0447S, Massachusetts, USA) and RNasin® Ribonuclease Inhibitor (Promega, #N2111, Wisconsin, USA) were then added to the reaction mixture, followed by incubation under the following conditions: 25 °C for 10 min, 37 °C for 1 h then 70 °C for 15 min.

Primers were designed for both human and rat *PCSK1*, and *GAPDH* as a loading control, as described in Table 1. Each PCR product, along with HyperLadder™ IV (Bioline, #BIO33030, TN, USA) as a molecular weight marker, was loaded onto a 2% agarose gel in 1× TAE and run at 100 V for 1 h, then imaged using a Gel Doc EZ Imager (Bio-Rad, #1708270, CA, USA).

**Table 1:** Primer sequences for amplification of PCSK1 and GAPDH.

Target	Primer	Primer sequence
PCSK1	Forward	5′-ATTCCAAAGTTGGAGGCATAAGAATG-3′
	Reverse	5′-TGTCTCCCCTGTTTGACACC-3′
Ghrelin	Forward	5′-GAGCCCTGAACACCAGAGAG-3′
	Reverse	5′-ACTGAACCCCTGACAGCTTG-3′
GAPDH	Forward	5′-CTCACGACCACAGTCCATGC-3′
	Reverse	5′-TTCAGCTCTGGGATGACCTT-3′

A relative qPCR was performed on the synthesised cDNA using the SensiFAST™ SYBR® No-ROX Kit (Bioline, #BIO-98020) on the Rotor-GeneTM 3000 (Corbett Research), with results normalised to GAPDH as a housekeeping gene.

### Induction of oxidative stress in vitro

To investigate the mechanisms of ghrelin protection, LV.LacZ or LV.hGhre was used to transduce H9c2s (MOI 100) or NRVMs (MOI 20). Four days post transduction, cells were injured with a dose titration of H_2_O_2_, whilst some cells were uninjured (0 µM H_2_O_2_) as a control. After 24 h, cell viability was assessed using the CellTiter 96® Non-Radioactive Cell Proliferation Assay (Promega, #G4000, WI, USA), hereafter abbreviated to “MTT assay”, according to manufacturer’s instructions.

### TMRE assay to assess mitochondrial function

Transduced H9c2 cells which had undergone injury with H_2_O_2_ were assessed together with uninjured control cells using the TMRE-Mitochondrial Membrane Potential Assay kit (abcam, #ab113852, Massachusetts, USA). Briefly, TMRE dye was added to each well at a final concentration of 200 nM, followed by incubation at 37 ^o^C for 30 min. Subsequently, cells were dissociated using trypsin, then washed in 2% FBS in PBS. Following staining with DAPI (0.1 mg/μL), cells were analysed for TMRE fluorescence using a Zeiss Axiovert 200 M live-cell imaging microscope (Zeiss, Oberkochen, Germany) and BD FACS Canto II (BD Biosciences, Franklin Lakes, NJ, USA).

### Flow cytometry

Cells were dissociated using TrypLE^TM^ Express, then washed with Dulbecco’s Phosphate Buffered Saline without calcium and magnesium. Cells were then stained using the Zombie NIR^TM^ Fixable Viability Kit (Biolegend, #423105, San Diego, USA). After washing, cells were stained with BV650 conjugated rat anti-CD90 antibody (Biolegend, #202533, San Diego, USA), then fixed in 4% PFA for 30 min, then washed and further stained with BV421 conjugated mouse anti-cTnT antibody (BD Biosciences, #565618, San Diego, USA) for 2 h. Upon further washing, cells were analysed on the FACSCanto^TM^ II Cell Analyzer or LSRFortessa^TM^ and data recorded using FACSDiva^TM^ Software (BD Biosciences, Franklin Lakes, NJ, USA). Analysis was subsequently performed using FlowJo (FlowJo LLC, Oregon, USA) version 10.

### Statistical analysis and software

Depending on the data, different statistical analyses detailed in the figure legends were performed using GraphPad Prism (GraphPad Software, La Jolla, CA, USA). All data are mean ± SEM. For all used tests, significance was represented as follows: *p < 0.05, **p < 0.01, ***p < 0.001, ****p < 0.0001. SnapGene software (www.snapgene.com) version 5.1.3 was used to design cloning strategies to demonstrate the rationale behind the two versions of the ghrelin vectors. Western blots and agarose gels were visualised using Image Lab software (Bio-rad Laboratories, Inc), version 6.1.0.

## Results

### Lentiviral mediated overexpression of human ghrelin in H9c2 cells

**Fig. 2:**
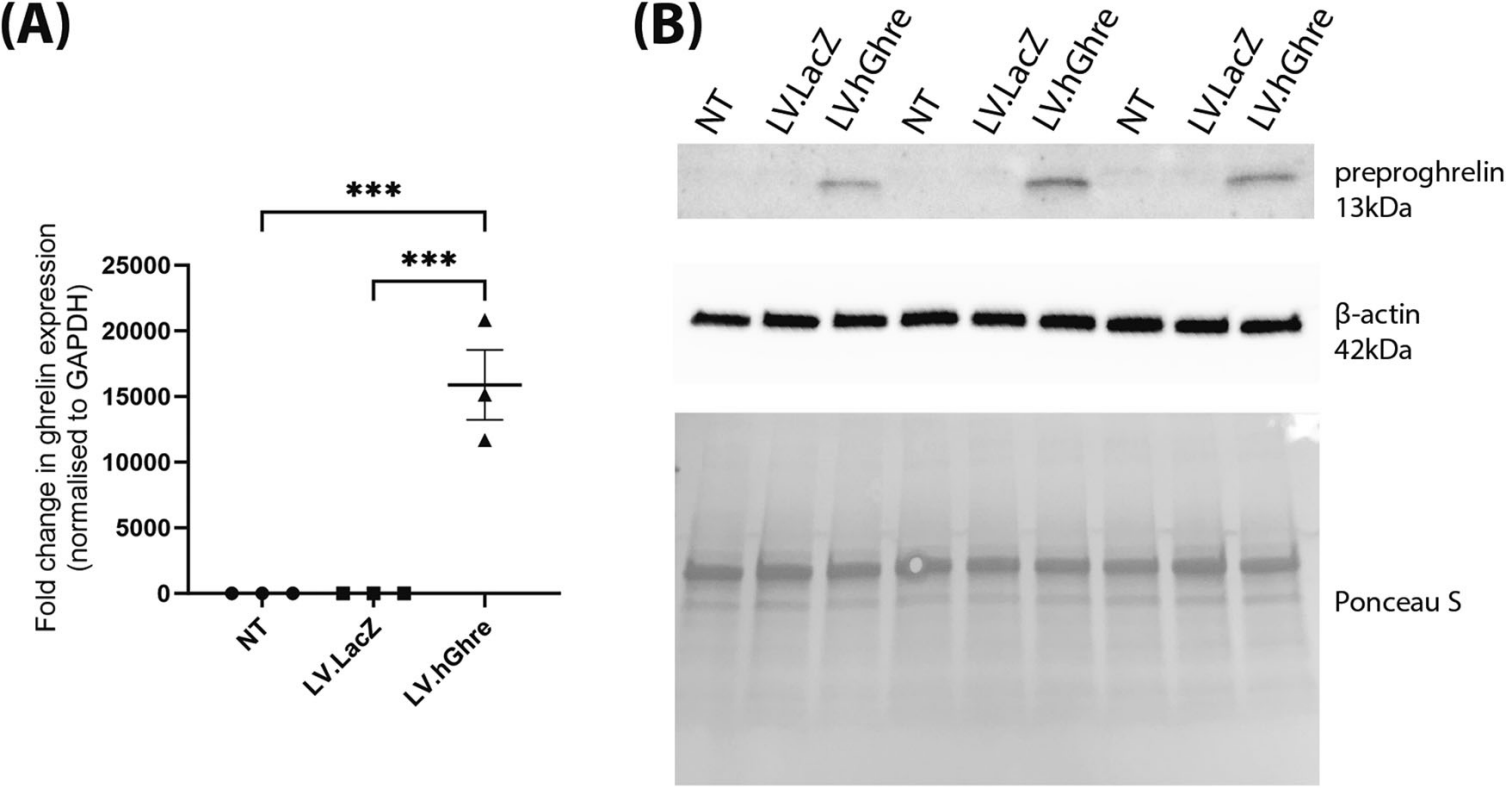
Recombinant human ghrelin was successfully overexpressed in H9c2 cells transduced with LV.hGhre. RNA and protein were extracted from H9c2 cells which were either non-transduced (NT), transduced with LV.LacZ (MOI 50) or transduced with LV.hGhre (MOI 50). **A** The relative fold change in ghrelin gene expression was quantified using qPCR and normalised to GAPDH (mean ± SEM, *n* = 3, ***p < 0.001). Analysis of significance was performed via one way ANOVA, with differences calculated using Tukey’s multiple comparison. **B** Protein blots from cell lysates were stained to detect ghrelin, β-actin and Ponceau S from H9c2 cells transduced with LV.LacZ or LV.hGhre, with NT as a negative control (*n* = 3).

To confirm overexpression of ghrelin, RNA was extracted from H9c2 cells transduced with LV.hGhre. RNA was also extracted from non-transduced (NT) H9c2s and cells transduced with LV.LacZ, as controls to compare ghrelin transgene expression. Based on relative qPCR analysis, H9c2 cells transduced with LV.hGhre had a 15,879 fold increase in ghrelin gene expression when compared to non-transduced cells and cells transduced with LV.LacZ (Fig. 2A). Statistical significance was reached when comparing ghrelin gene expression in non-transduced cells and H9c2 cells transduced with LV.hGhre. There was also an increase in ghrelin protein which could be detected by western blot from the cell lysate (Fig. 2B). This showed successful gene delivery to the H9c2 cells resulted in detectable overexpression of the ghrelin protein.

### Overexpression of ghrelin did not protect H9c2 cells from H_2_O_2_ injury

**Fig. 3:**
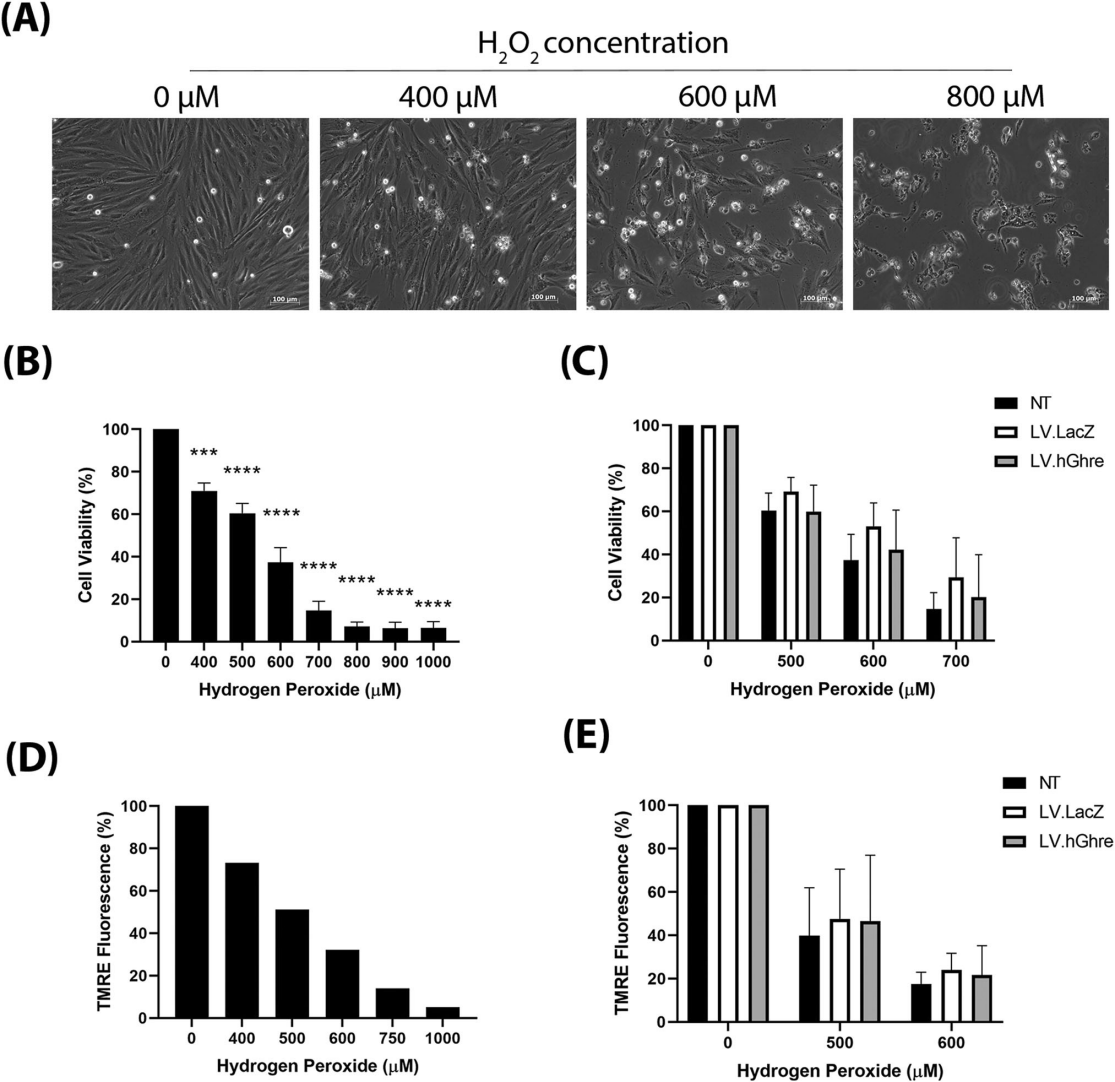
H9c2 cells were not protected against H_2_O_2_ injury, despite overexpression of ghrelin. H9c2 cells were treated with H_2_O_2_ for 24 h then cell viability was assessed using the MTT assay. **A** Morphological changes of H9c2 cells following treatment with H_2_O_2_ (scale bar = 100 µm). **B** Mean H9c2 cell viability following treatment with H_2_O_2_, expressed as a percentage of the non-treated 0 µM H_2_O_2_ control (mean ± SEM, *n* = 3, ***p < 0.001, ****p < 0.0001). This was analysed with one way ANOVA using Dunnett’s multiple comparisons. **C** Data represents the mean cell viability of non-transduced (NT), LV.LacZ transduced (MOI 100) and LV.hGhre transduced (MOI 100) H9c2 cells following treatment with H_2_O_2_, expressed as a percentage of the non-treated 0 µM H_2_O_2_ control (mean ± SEM, *n* = 3). This was analysed using two way ANOVA with Tukey’s multiple comparison. **D** Data represents the H9c2 TMRE fluorescence following treatment with H_2_O_2_, expressed as a percentage of the non-treated 0 µM H_2_O_2_ control (*n* = 1). **E** Data represents the mean H9c2 TMRE fluorescence of NT, LV.LacZ transduced and LV.hGhre transduced H9c2 cells following treatment with H_2_O_2_, expressed as a percentage of the non-treated 0 µM H_2_O_2_ control (mean ± SEM, *n* = 3). This was analysed using two way ANOVA with Tukey’s multiple comparison.

We then set out to determine whether ghrelin overexpression was cardioprotective in the context of oxidative stress. Firstly, to establish an appropriate dose of H_2_O_2_ to treat H9c2 cells with, a range of concentrations (0–1000 µM) were added to the cells and morphological changes were observed. H9c2 cells which were not treated with H_2_O_2_ (0 µM) exhibited a spindle shape morphology (Fig. 3A). As the concentration of H_2_O_2_ increased, the H9c2 cells became more rounded and, eventually at 800 µM H_2_O_2_, very few cells were adhered to the plate and floating rounded dead cells could be observed. The H9c2 cell viability at a range of concentrations of H_2_O_2_ (0–1000 µM) was quantified using the MTT assay.

A general trend of decreasing cell viability with increasing H_2_O_2_ concentration was observed (Fig. 3B). Based on this result, concentrations of 500, 600 and 700 µM H_2_O_2_ were chosen to use for subsequent experiments involving cell viability, since each concentration decreased mean cell viability to 60%, 37% and 15%, respectively (Fig. 3B), which encompassed a broad range to be able to observe whether ghrelin overexpression causes protection.

Cell viability was assessed in non-transduced (NT), LV.LacZ transduced and LV.hGhre transduced H9c2 cells treated with 500, 600 and 700 µM H_2_O_2_. Cells were transduced at MOI 100, based on a vector dose titration using LV.GFP (Supplementary Fig. 2A). No significant change in viability was observed across all the treatment groups (Fig. 3C). Hence, it was concluded that LV.hGhre did not increase cell viability following H_2_O_2_ injury when compared to the LV.LacZ control.

To further explore the ability of LV.hGhre to protect H9c2 cells from H_2_O_2_ injury, mitochondrial membrane potential was assessed using the TMRE assay. H9c2 cells which were not treated with H_2_O_2_ (0 µM), exhibited bright red TMRE fluorescence (Fig. 3D). However, as the concentration of H_2_O_2_ increased, the cells exhibited less TMRE fluorescence, which was quantified using flow cytometry (Fig. 3D).

Based on this result, concentrations of 500 and 600 µM H_2_O_2_ were chosen to use for subsequent experiments exploring H9c2 mitochondrial function. Mitochondrial membrane potential was evaluated in non-transduced (NT), LV.LacZ transduced and LV.hGhre transduced H9c2 cells treated with 500 and 600 µM H_2_O_2_. Both LV.LacZ and LV.hGhre transduced H9c2 cells exhibited increased TMRE fluorescence when compared to non-transduced cells at both 500 and 600 µM H_2_O_2_ (Fig. 3E). However, LV.hGhre transduced cells did not show increased TMRE fluorescence when compared to LV.LacZ transduced cells at both H_2_O_2_ concentrations. Hence, it was concluded that LV.hGhre did not protect against H_2_O_2_ induced mitochondria damage.

### Mature ghrelin was not produced in H9c2 cells due to absence of PCSK1

**Fig. 4:**
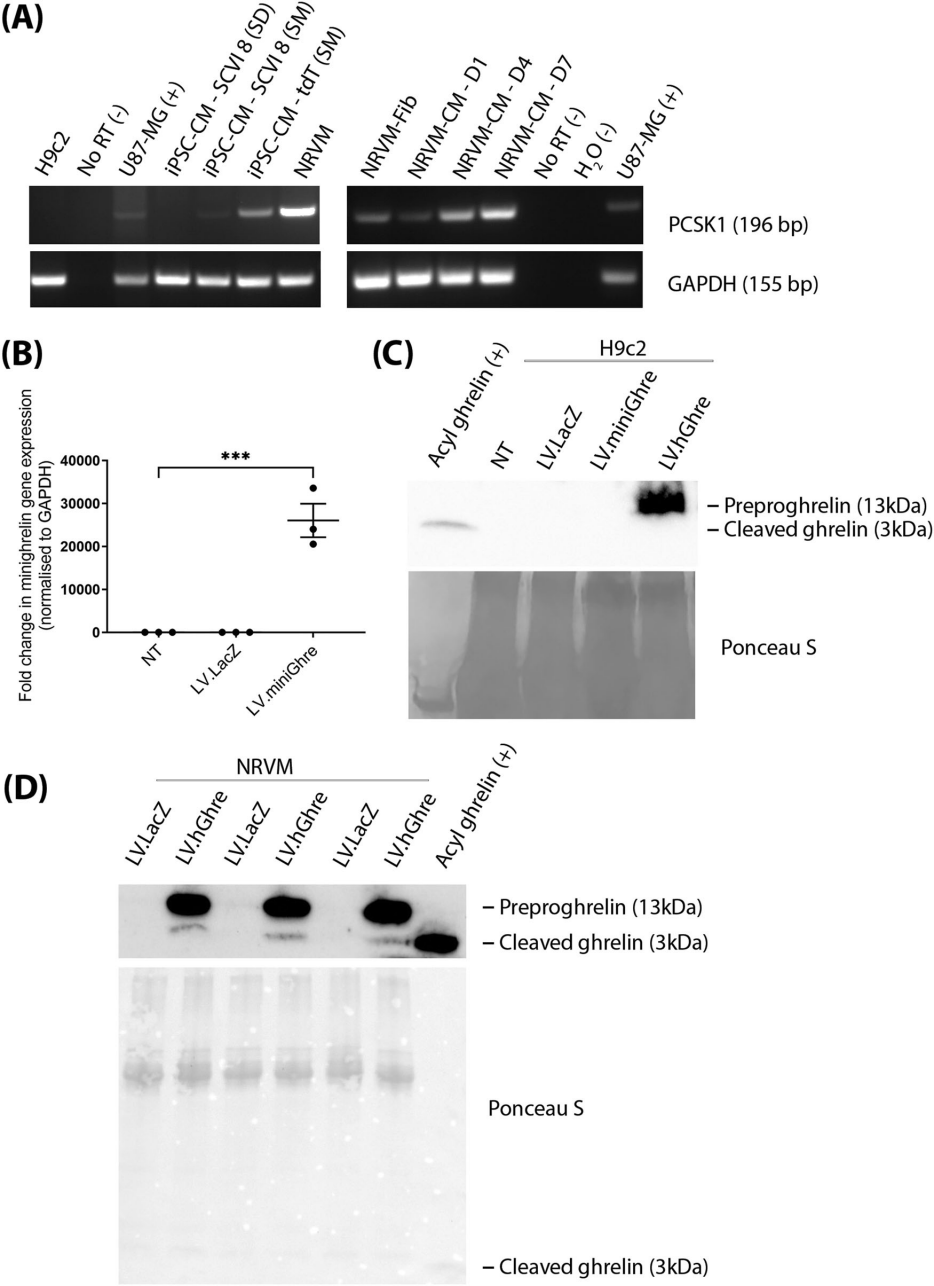
*PCSK1* expression is required for processing of preproghrelin to mature ghrelin. **A** *PCSK1* expression (196 bp) in H9c2s, iPSC-CMs and NRVMs at different times of culture, with U87-MG as a positive control, and No Reverse Transcriptase sample (RT) and water as negative control. GAPDH (155 bp) was used as a loading control. **B** Relative fold change in H9c2 minighrelin expression was quantified using qPCR and normalised to GAPDH (mean ± SEM, *n* = 3, ***p < 0.001). This was analysed with one way ANOVA using Dunnett’s multiple comparisons **C** Immunoblot of ghrelin in the supernatant of H9c2s transduced with LV.LacZ, LV.miniGhre, LV.hGhre (MOI 100). **D** NRVMs transduced with LV.LacZ or LV.hGhre at MOI 20. Immunoblots had acyl ghrelin (250 ng) loaded as a control for the size of mature cleaved ghrelin protein (3 kDa, shown with yellow arrows). Blots were also stained with Ponceau S as the loading control.

After observing that LV.hGhre, despite causing ghrelin overexpression, failed to maintain H9c2 cell viability and mitochondrial function following H_2_O_2_ injury, it was decided to explore potential reasons for this outcome. One possible reason was that, in H9c2 cells, preproghrelin was unable to be cleaved and processed into the mature biologically active forms of ghrelin (Fig. 1). Hence, it was decided to explore whether H9c2 cells express the preproghrelin cleavage enzyme prohormone convertase 1/3 (PC1/3), encoded by the gene *PCSK1*. We also explored whether other cell types would exhibit more appropriate expression of *PCSK1*. To do so, RNA was extracted from H9c2 cells and hiPSC-CMs, followed by cDNA synthesis and PCR amplification of the *PCSK1* gene. The U87-MG cells were included as a positive control, since *PCSK1* is known to be highly expressed in the brain [22]. No signal was observed from the H9c2 cell sample, confirming that these cells do not express the PCSK1 gene (Fig. 4A).

To become biologically active, preproghrelin must be processed to mature forms of ghrelin during translation and subsequently secreted from the producing cell. Hence, it was decided to explore whether it was possible to generate a ghrelin transcript which would not require cleavage by PC1/3 to generate active protein. H9c2 cells transduced with the truncated LV.miniGhre vector showed overexpression of the transgene by qPCR (Fig. 4B).

We also aimed to determine whether mature ghrelin was secreted into the supernatant of cells transduced with LV.miniGhre and LV.hGhre. Supernatant was collected from transduced cells then concentrated, followed by Western blot analysis. Mature acyl ghrelin protein was used as a positive control which appeared at 3 kDa. However, this 3 kDa ghrelin protein could not be detected by western blot in both H9c2 cells transduced with LV.miniGhre and LV.hGhre. (Fig. 4C). Hence, it was concluded that mature ghrelin is not secreted into the supernatant of these transduced H9c2 cells. Interestingly, a band which was ~13 kDa, the same molecular weight of preproghrelin protein, was present in the supernatant of cells transduced with LV.hGhre (Fig. 4C).

We also tested whether mature ghrelin was produced from transduced NRVMs. Mature ghrelin was evident in the supernatant of NRVMs transduced with LV.hGhre, although this was substantially overshadowed by uncleaved preproghrelin (Fig. 4D). From these results, we concluded that NRVMs were able to generate functional ghrelin, most likely due to the presence of PC1/3 encoded by *PCSK1*, which allowed cleavage of preproghrelin into the biologically active ghrelin.

### PCSK1 is expressed in other cardiac cell types

Other cardiac cell types were explored for use in this project. Interestingly, NRVMs, which are primary rat ventricular myocytes, appeared to express *PSCK1*, with expression maintained in culture over 7 days (Fig. 4A). The increase in *PCSK1* is correlated to a decrease in purity of cardiomyocytes over time in culture (Supplementary Fig. 1A–C). At D14 of culture, the NRVMs were predominantly fibroblasts (Supplementary Fig. 1D). *PCSK1* expression was not seen in hiPSC-CMs which were differentiated using the commercially available stem diff cardiomyocyte differentiation kit (Fig. 4A). Intriguingly, *PCSK1* expression was observed from the same stem cell line differentiated using the small molecule protocol [23, 24]. This seems to imply that the cardiac cells resulting from the two differentiation methods may be phenotypically distinct with regard to *PSCK1* expression.

### Ghrelin overexpression was cardioprotective in NRVMs that expressed PCSK1

**Fig. 5:**
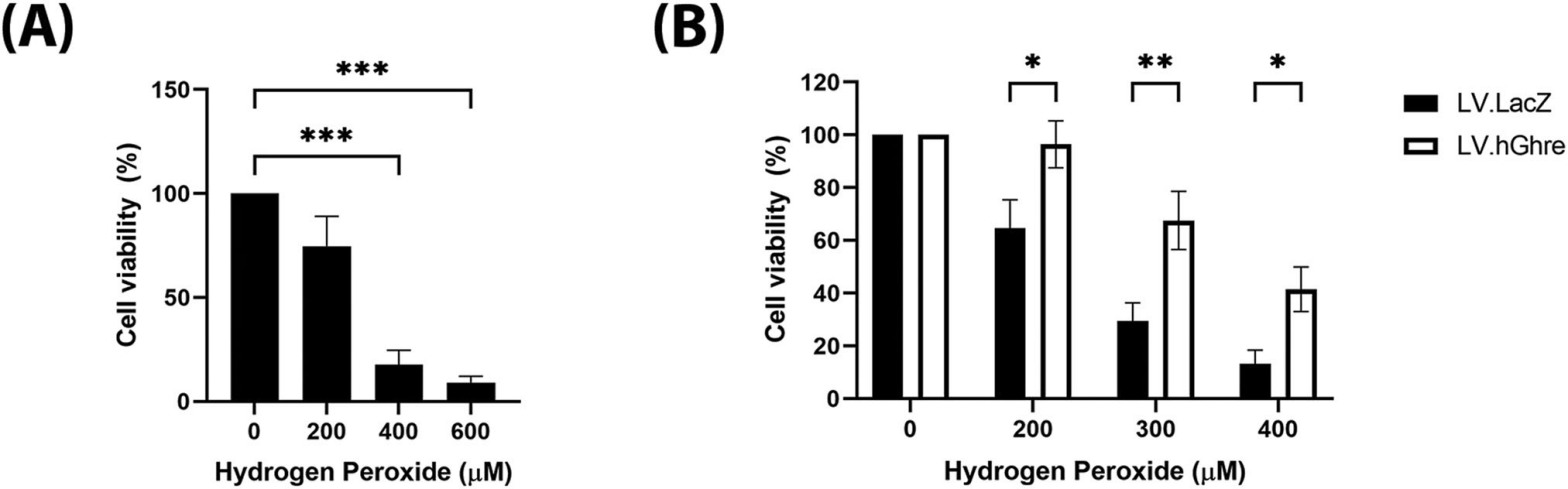
Ghrelin mediated protection against H_2_O_2_ in NRVMs. NRVMs were treated with H_2_O_2_ for 24 h then viability was assessed using the MTT assay. **A** Data represents the mean NRVM cell viability expressed as a percentage of the non-treated 0 µM H_2_O_2_ control (mean ± SEM, *n* = 3, ***p < 0.001). This was analysed with one way ANOVA using Dunnett’s multiple comparisons. **B** Data represents the mean cell viability of LV.LacZ transduced (MOI 20) and LV.hGhre transduced (MOI 20) NRVM cells following treatment with H_2_O_2_, expressed as a percentage of the non-treated 0 µM H_2_O_2_ control (mean ± SEM, *n* = 10, *p < 0.05, **p < 0.01). This was analysed using two way ANOVA with Sidak’s multiple comparison.

Cell viability was then assessed in NRVMs that were transduced with LV.hGhre at MOI 20. The vector dose required to transduce these cells was determined by titration of LV.GFP (Supplementary Fig. 2B). To establish an appropriate dose of H_2_O_2_ to treat NRVMs, a range of concentrations (0 – 600 µM) were added to the cells, with viability quantified using the MTT assay. A dose dependent decrease in cell viability with increasing H_2_O_2_ concentration was observed, with statistical significance being reached for cells treated with 400 and 600 μM when compared to the non-treated 0 µM H_2_O_2_ control (Fig. 5A). Based on this result, concentrations of 200, 300 and 400 µM H_2_O_2_ were chosen to use for subsequent experiments involving cell viability. Viability was improved in the NRVMs transduced with LV.hGhre compared to LV.LacZ at all concentrations of H_2_O_2_ tested (Fig. 5B). Thus, we proved that functional ghrelin produced from the transduced NRVM culture was protective against H_2_O_2_ mediated oxidative stress.

### Ghrelin overexpression was not protective in hiPSC-CM, despite expression of PCSK1

**Fig. 6:**
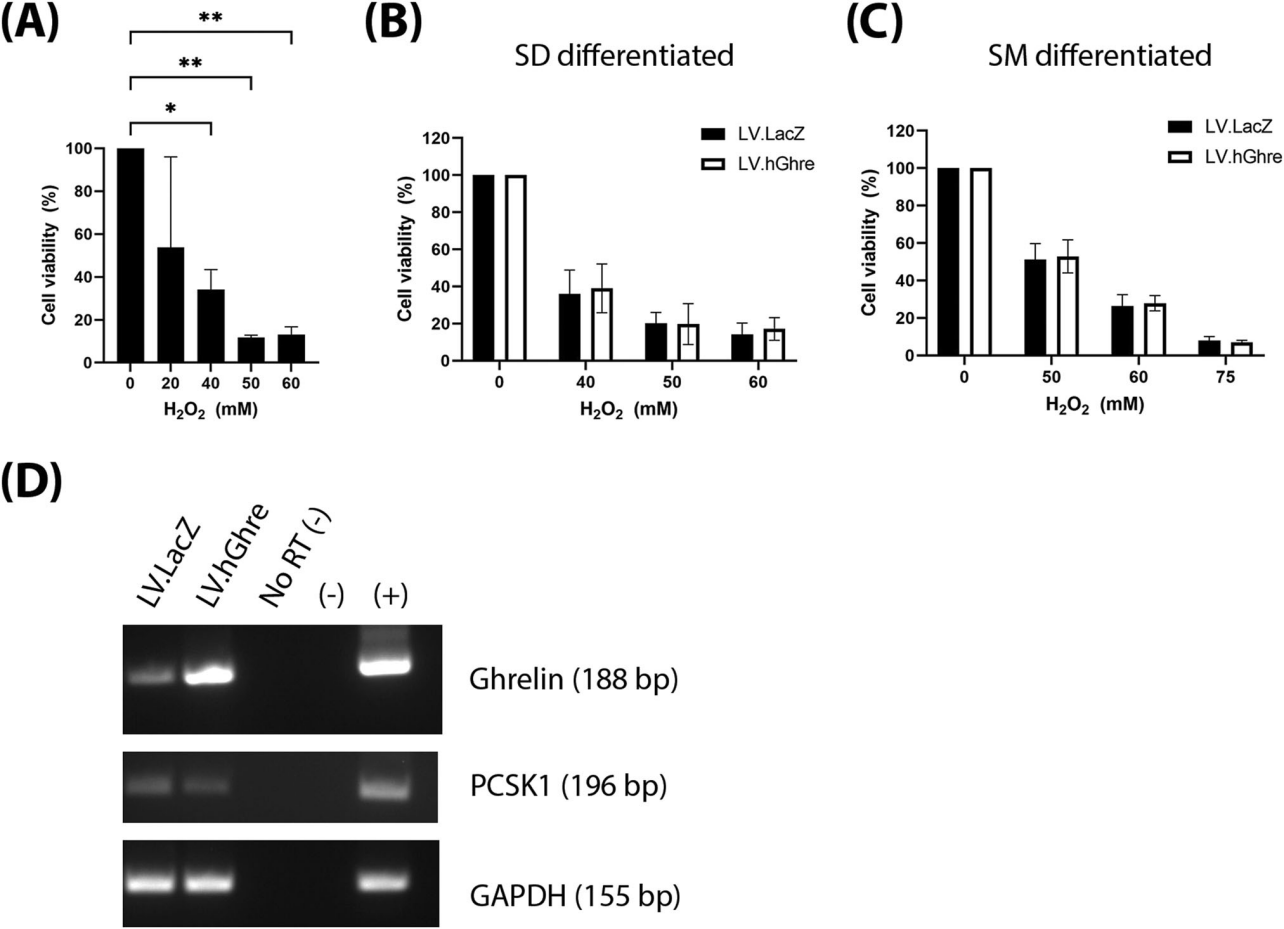
Ghrelin was not protective against H_2_O_2_ induced oxidative stress in hiPSC-CMs. hiPSC-CMs transduced with LV.hGhre (MOI 20) or LV.LacZ (MOI 20) were treated with H_2_O_2_ for 24 h then viability was assessed using the MTT assay. **A** Kill curve showing viability in hiPSC-CMs expressed as a percentage of the 0 µM H_2_O_2_ control. This was analysed with one way ANOVA using Dunnett’s multiple comparisons. **B** Data represents the mean cell viability of transduced hiPSC-CMs differentiated with the STEMDIFF (SD) protocol, expressed as a percentage of the 0 µM H_2_O_2_ control (mean ± SEM, *n* = 4, ***p < 0.001). This was analysed using two way ANOVA with Sidak’s multiple comparison. **C** Data represents the mean cell viability of transduced hiPSC-CMs differentiated with the small molecule (SM) protocol, expressed as a percentage of the 0 µM H_2_O_2_ control (mean ± SEM, *n* = 3, *p < 0.05, **p < 0.01). This was analysed using two way ANOVA with Sidak’s multiple comparison. **D** Gel image of PCR amplicons for ghrelin, *PCSK1* and GAPDH from hiPSC-CMs transduced with LV.LacZ or LV.hGhre.

After observing the protective effect of ghrelin in NRVMs, we then explored whether the same therapeutic effect was possible in hiPSC-CMs. These cells were transduced at MOI 20, with the dose chosen based on a vector titration of LV.GFP (Supplementary Fig. 2C). To establish an appropriate dose of H_2_O_2_ to treat hiPSC-CMs with, a range of concentrations (0–60 mM) were added to the cells, with viability quantified using the MTT assay. A dose dependent decrease in cell viability with increasing H_2_O_2_ concentration was observed (Fig. 6A). Based on this result, concentrations of 50, 60 and 75 mM H_2_O_2_ were chosen to use for subsequent experiments involving cell viability.

Considering the previous observed results in NRVMs, we hypothesised that protection would be seen in *PCSK1* expressing SM differentiated hiPSC-CMs, while there would be no benefit in SD differentiated cells which lacked *PCSK1* (Fig. 4A). However, we observed that viability was not improved in the hiPSC-CMs transduced with LV.hGhre compared to LV.LacZ at all concentrations of H_2_O_2_ tested, regardless of the differentiation by SD or SM protocol (Fig. 6B, C, respectively). This was surprising in the case of the SM differentiated cells, as we confirmed by PCR the successful overexpression of ghrelin in the presence of *PCSK1* (Fig. 6D).

## Discussion

In this manuscript, we focused on evaluating the effect of ghrelin in the hydrogen peroxide injury model. Lentivector expressed ghrelin was able to confer protection from oxidative stress on primary cardiac cells with endogenous *PCSK1* expression (NRVMs) but not in cell lines regardless of *PCSK1* expression (H9c2 and hiPSC-CMs). The role of *PCSK1* is critical as it led to processing of the preproghrelin to a secreted, biologically active form of ghrelin as demonstrated in the supernatant of NRVMs. This becomes a rate limiting step, as low level of PCSK1 expression would then lead to low levels of PC1/3, which is required to cleave preproghrelin. The conflicting results in ghrelin mediated protection between hiPSC-CMs and NRVMs may be due to differences in the maturity between the two cell types, perhaps then leading to lower expression levels of PCSK1 in the hiPSC-CMs. Variation in gene expression has been observed when comparing cell cultures derived from separate differentiation protocols [25]. This may be due to differences in the rate of maturation of the cardiomyocytes differentiated from separate protocols, though it does not preclude the possibility of subtle phenotypical differences in cells generated from distinct protocols. In this study, the variation in PCSK1 expression from cells generated using the SD and SM protocols may suggest differences in the cardiomyocyte populations. It is also possible that the SM protocol has generated a distinct non-myocyte population which exhibits higher expression of PCSK1. Further validation is required to determine which population of cells is expressing PCSK1, with functional PC1/3 activity.

We showed that LV.hGhre caused ghrelin overexpression in NRVMs with subsequent transcription, translation and secretion of ghrelin protein into the cell culture media (Fig. 4). However, there were two ghrelin band sizes in NRVM supernatant. A dominant 10–15 kDa band, likely representing preproghrelin and a 3 kDa faint band corresponding to the active ghrelin variant. It is possible that the NRVMs that were highly overexpressing ghrelin were dying in culture and releasing the uncleaved preproghrelin protein into the culture supernatant. It is also possible that the rate of protein cleavage by PC1/3 was not able to match the levels of overexpression in the cells. To clarify this, in future work, NRVM expression of the cleavage enzymes signal peptidase and prohormone convertase 1/3 may be more closely explored. *PCSK1* could be knocked down with siRNA to observe if there are subsequent inhibitory effects on LV.hGhre mediated cardioprotection

Despite the apparent low expression of mature ghrelin in NRVM supernatant, LV.hGhre was shown to significantly increase NRVM cell viability following injury with H_2_O_2_, compared to the LV.LacZ controls. Based on other studies, such as the 2011 study showing that ghrelin administration decreased H_2_O_2_ induced cell death of primary rat oligodendrocytes [26], this result was expected.

Our study shows that for ghrelin gene therapy to be effective, the vector needs to deliver the transgene to cells which are able to cleave preproghrelin to the active ghrelin peptide. The mature ghrelin protein can then be secreted and exert a paracrine protective effect on nearby cells. It was interesting that *PCSK1* expression increased as the NRVMs were cultured over 7 days. We have found that the purity of NRVM culture decreases with time, and that fibroblasts can outgrow cardiomyocytes by 1–2 weeks (Supplementary Figure 1). We hypothesised that while the transduced culture contained both myocytes and fibroblasts mainly, it is possible that the mature ghrelin is synthesised more efficiently from non-myocyte populations.

A challenge in the use of ghrelin as a cardioprotective therapy is the need for temporal and spatial restriction of its activity to the site of injury. This may be partially addressed with the use of ghrelin peptides, which are transiently present, yet able to induce cardioprotective effects. However, depending on the disease being treated, the transient activity of the peptide may mean that re-administration becomes necessary. It is also difficult to then localise treatment to the heart, if administration is systematic.

It is currently unknown if there are off-target effects resulting from ghrelin overexpression outside of the heart. Although ghrelin is known as the hunger hormone, transgenic mouse models overexpressing ghrelin did not show the adverse effect of increased weight gain despite elevated levels of circulating ghrelin [27, 28]. It was hypothesised that this may be due to a paradoxical increase in energy expenditure, as animals were shown to have increased locomotion, in anticipation of feeding [29, 30]. Other gene therapy methods such as adeno-associated virus vectors or custom designed lipid nanoparticles may allow targeting of gene delivery to cardiac cells. However, this would need to be used in conjunction with a strategy to regulate expression of ghrelin so that it is only upregulated when required to reduce oxidative stress. Each strategy has its own set of challenges to address, and further work may explore the use of these methods as alternative methods for delivery of ghrelin to the heart.

## Limitations of the study

Although our aim was to determine the antioxidant mediated protection of ghrelin, we could only show this in the NRVM cultures. As it was not possible to show this in primary human cells, our alternative was to use hiPSC-CMs. However, these cell lines are relatively immature in comparison to primary cardiomyocytes, and may not therefore have the same mechanisms for processing of preproghrelin to the mature biologically active peptide. Further work would be required to test the potential of this therapy in vivo, as the models we have currently used do not allow us to evaluate off-target effects that may occur.

We have also used *PCSK1* detection by PCR to infer the presence of PC1/3, which is required to cleave preproghrelin into acyl ghrelin. We did not therefore confirm the expression or activity of PC1/3 directly in the cells tested. This may be something that can be addressed in future studies to further explain why ghrelin gene therapy was not protective in hiPSC-CMs.

Lentiviral vectors which integrate into the host genome have limited application in clinical settings. Gene dysregulation may occur at random sites of integration into the host cell genome, which may cause cancer by activating oncogenes or inactivating tumour suppressor genes [31]. Also, since most lentiviral vectors are derived from HIV-1, there are theoretical concerns a functional pathogenic virus may be reconstituted [31]. There have been clinical trials using lentiviral vectors in haemopoietic stem cells to correct haemoglobin deficiencies which have had no adverse events [32], however, the long term implications are unclear. Hence, moving forward with in vivo studies, it may be more feasible to use AAV vectors which have a higher safety profile [33].

## Conclusions

We have shown that ghrelin gene therapy can be used as a cardioprotective strategy which can protect cardiomyocytes from oxidative stress. Ghrelin expression is able to protect NRVMs from H_2_O_2_ injury, but not in other studied cell types.

### Supplementary information

Supplementary figure legends

Supplementary Figure 1

Supplementary Figure 2

Supplementary Figure 3

## Data Availability

All data generated or analysed during this study are included in this published article and its supplementary information files. The data presented are available from the corresponding author on request.
