# Supplementary material for: Ghrelin mediated cardioprotection using in vitro models of oxidative stress
Source: Gene Ther. 2024 Jan 4;31(3-4):165–74. doi: 10.1038/s41434-023-00435-9 (PMC10940144; doi:10.1038/s41434-023-00435-9)
Supplement: Supplementary file 1 — Supplementary figure legends [file 41434_2023_435_MOESM1_ESM.docx]

**Supplementary Figure legends**

**Supplementary Figure 1: Decrease in cardiomyocyte purity over time in cultures of NRVMs.** Cardiomyocyte purity was assessed in NRVMs by flow cytometry at Week 0 (day 1), 1 and 2 post isolation. (A) NRVMs were imaged prior to flow cytometry under a light microscope (n=2). (B) Gating strategy for flow analysis of NRVMs stained with cTnT as a marker for myocytes at Week 0 (C) The purity of NRVM preps expressed as cTnT+ cells within the live cell population, shown over two weeks of culture. (D) Gating strategy to quantify the cTnT+ and CD90+ cell populations in the NRVM prep at D14.

**Supplementary Figure 2: Optimisation of vector dose required to transduce cardiac cells with lentivirus *in vitro*.** Cells were transduced with a range of MOIs using LV.GFP to assess transduction efficiency. After four days, the cells were imaged under a fluorescent microscope. Cells were then harvested and assessed for GFP+ percentage by flow cytometry. (A) H9c2 MOI range was 0, 20, 100, 200 (± SEM, n=3). (B) NRVM MOI range was 0, 1, 10, 20 (mean ± SEM, n=7). (C) hiPSC-CM MOI range was 0, 1, 10, 20 (± SEM, n=3). *p<0.05, **p<0.01, ***p<0.001, ****p<0.0001. These data were analysed by using two way ANOVA with Dunnett’s multiple comparison.

**Supplementary Figure 3: Design rationale of the truncated “minighrelin”.** The regions encoding for downstream peptide products from the 351 bp human ghrelin mRNA (NM_001134941.3) were determined (mat peptide = mature peptide). To generate a ghrelin peptide product which would not require PC1/3 mediated cleavage to generate biologically protein, the transgene was truncated to encompass only the region that encoded for the signal peptide and the ghrelin mature peptide.
