# Supplementary figures and images for: Ghrelin mediated cardioprotection using in vitro models of oxidative stress
Source: Gene Ther. 2024 Jan 4;31(3-4):165–74. doi: 10.1038/s41434-023-00435-9 (PMC10940144; doi:10.1038/s41434-023-00435-9)

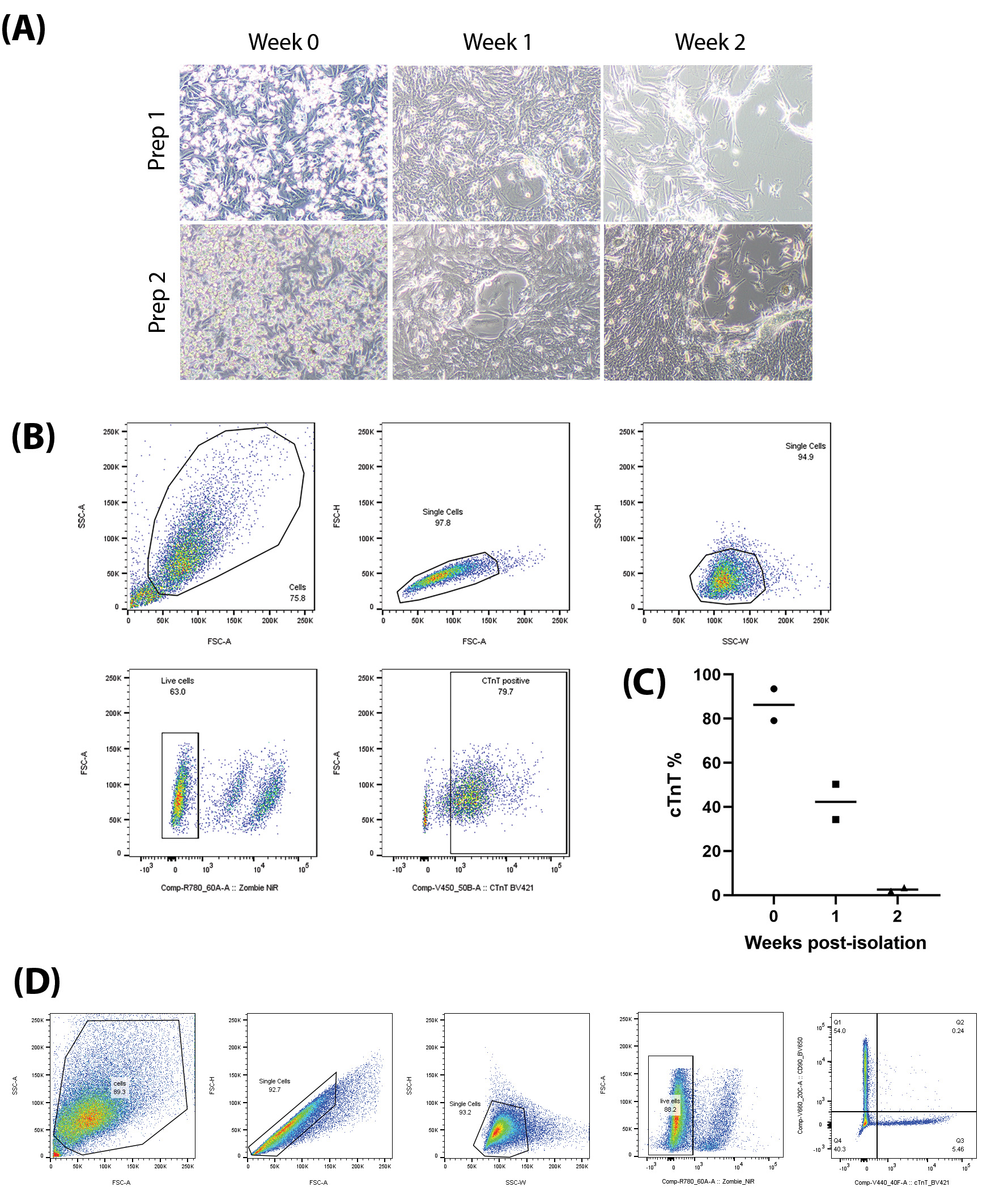

Supplement: Supplementary file 2 — Supplementary Figure 1 [file 41434_2023_435_MOESM2_ESM.jpg]

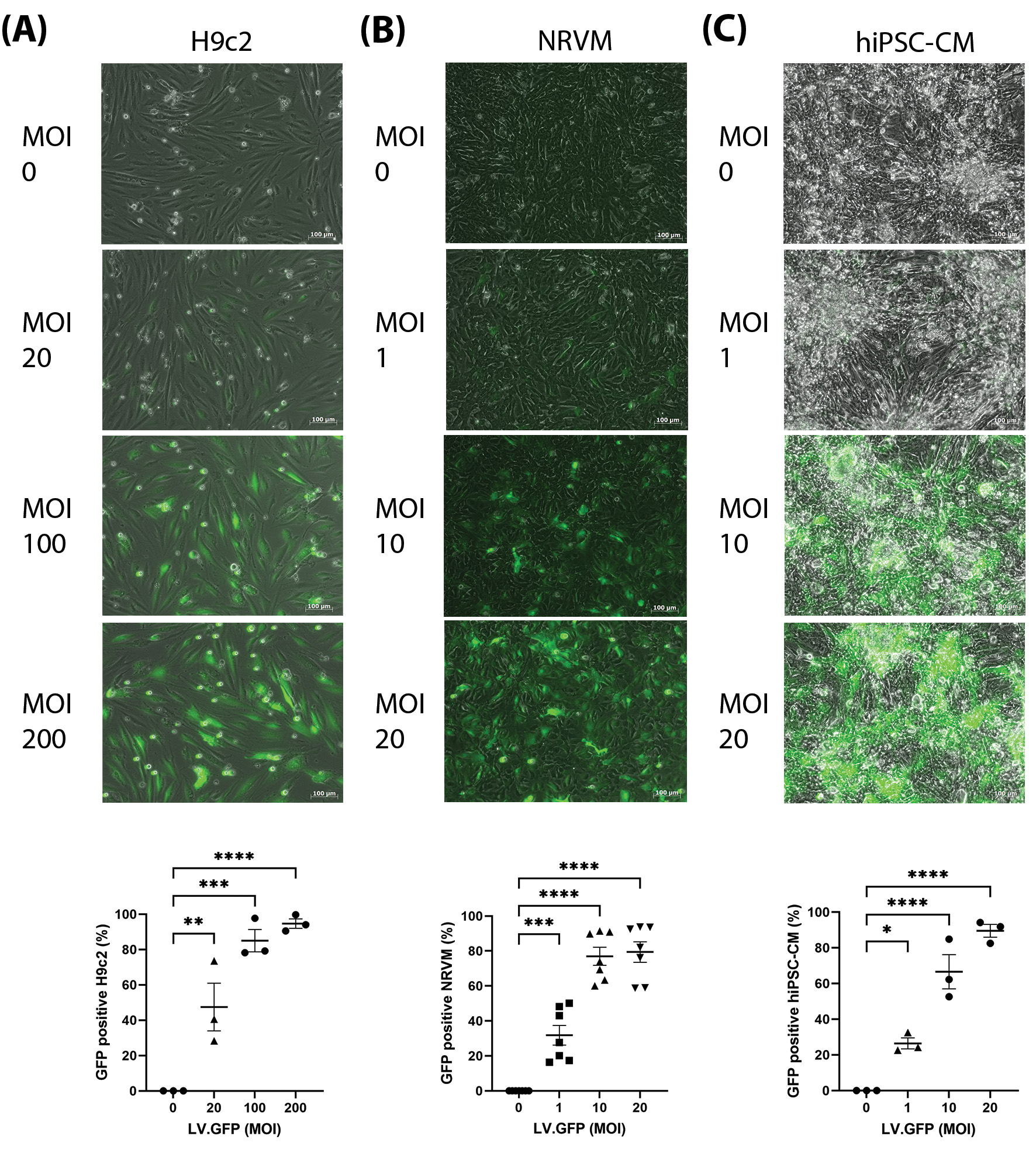

Supplement: Supplementary file 3 — Supplementary Figure 2 [file 41434_2023_435_MOESM3_ESM.jpg]

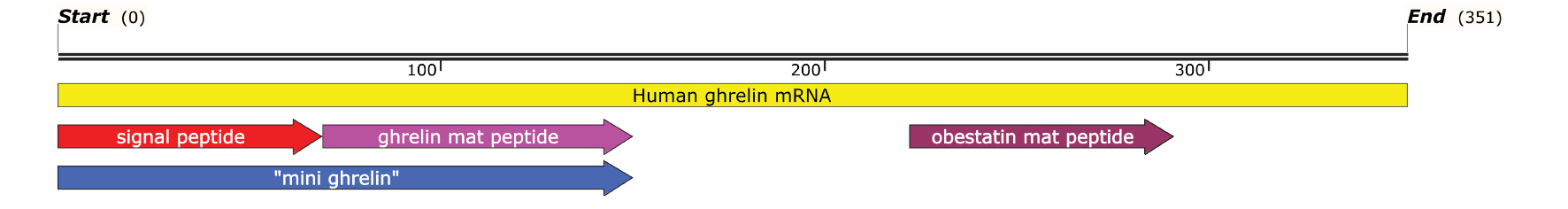

Supplement: Supplementary file 4 — Supplementary Figure 3 [file 41434_2023_435_MOESM4_ESM.jpg]
